# Post-discharge pharmacological treatment discontinuation of forensic psychiatric patients in Sweden

**DOI:** 10.3389/fpsyt.2024.1342722

**Published:** 2024-02-09

**Authors:** Ebba Noland, Suvi Virtanen, Fia Klötz Logan, Zheng Chang, Mattias Strandh

**Affiliations:** ^1^ Department of Social Work, Umeå University, Umeå, Sweden; ^2^ Sundsvall Forensic Psychiatric Centre, Region Västernorrland, Sundsvall, Sweden; ^3^ Department of Medical Epidemiology and Biostatistics, Karolinska Institutet, Stockholm, Sweden

**Keywords:** forensic psychiatry, mentally disordered offender, medication discontinuation, pharmacological treatment discontinuation, psychotropic medications, discharge

## Abstract

**Introduction:**

Most forensic psychiatric patients have chronic psychiatric disorders that require long-term pharmacological treatment even after discharge from care. However, the prevalence and correlates of post-discharge medication discontinuation in this patient group remain unclear.

**Objective:**

The aim of this study was to investigate the prevalence and correlates of post-discharge discontinuation of pharmacological treatment in forensic psychiatric patients in Sweden.

**Methods:**

Data on individuals discharged from forensic psychiatric care between 2009 and 2018 (*n* = 1,142) with ongoing pharmacological treatment at the time of discharge (*n* = 856) were identified from the Swedish National Forensic Psychiatric Register. Cox regression models were used to estimate the association between patient characteristics and medication discontinuation.

**Results:**

Of the 856 individuals with pharmacological treatment at discharge, 488 (57%) discontinued treatment within 2 years of discharge. Factors associated with an increased risk of treatment discontinuation varied between different types of psychotropic medications: the most important correlate was comorbidity between psychosis and personality disorder. Higher age at discharge, longer length of stay, having a history of several psychiatric care episodes, having a trustee, having a limited guardian, and a residing in a supported living accommodation at the time of discharge were associated with a decreased rate of medication discontinuation. This applied for antipsychotics, antidepressants, antiepileptics, and any psychotropic medication, but not for psychostimulants or drugs used in addictive disorders.

**Conclusion:**

For many former forensic psychiatric patients, there are situational factors associated with medication discontinuation. This insight holds significance for professionals who are involved in pre-discharge planning within forensic psychiatric care and those who interact with this cohort of former patients post-discharge.

## 1 Introduction

When a patient is discharged from forensic psychiatric care, clinicians’ responsibility for their wellbeing concludes, along with their access to information regarding the patient’s ongoing condition. In Sweden, a majority of forensic psychiatric patients have a psychotic disorder, predominantly schizophrenia (1), a condition for which the need for medication is often lifelong (2). Swedish legislation (3) concerning discharge from forensic psychiatric care states that several different aspects need to be taken into consideration before a decision about discharge can be made. This includes the mental state of the patient, the risk of criminal recidivism, and other personal conditions. However, no comprehensive evaluation regarding the continuity of pharmacological treatment in this group exists.

Previous studies have revealed a nonadherence rate of 43% to prescribed medications among non-forensic psychiatric outpatients (4). Post-discharge, 68% of general psychiatric patients discontinued their psychiatric medication, and 25% discontinued using antipsychotics within 3 months after discharge (5). Research focusing only on individuals with schizophrenia indicated that as many as 74% of the group discontinued the specific type of pharmacological treatment within 18 months (6). Additionally, within 1 year of hospital discharge, 26% discontinued medication (7). Despite the potential effectiveness of these medications, treatment discontinuation presents great challenges for the treatment of patients with chronic psychotic disorders. Adherence to antipsychotics is especially important, as non-adherence has been shown to be associated with a highly elevated risk of suicide (8), among other poor outcomes. Consequently, clinicians exert considerable efforts to enhance adherence to pharmacological treatment at the time of discharge.

Factors associated with treatment persistence can be categorized into four areas: patient-related, psychological, medication-related, and social/environmental (9). Patient-related factors encompass aspects such as age, sex, and educational level. For instance, there is an association between lower age and medication discontinuation in patients with psychotic illness (10). An example of a psychological factor is a negative attitude toward their ongoing medication, while medication-related factors could refer to side effects. Social and environmental factors encompass stability of living arrangement, supervision of pharmacological administration, and lack of family support. As mentally disordered offenders in Sweden are a distinct group often residing in supported living accommodations post-discharge from forensic psychiatric services (11), the prevalence of these risk factors for medication discontinuation may differ from the general population.

Treatment persistence also holds significant implications for the risk for criminal recidivism, a finding supported by extensive research across diverse groups. Studies investigating the impact of antipsychotics and mood stabilizers on violent crime (12, 13) have consistently shown a strong association between medication adherence and a reduction in violent crime within the broader population. Regarding individuals released from prison, Chang et al. (14) came to a similar conclusion: periods of psychotropic medication adherence were associated with lower rates of violent reoffending compared to non-adherence periods in this cohort. Among offenders with diagnosed schizophrenia, good treatment persistence to antipsychotic medication demonstrated reductions in both violent and non-violent offending (15, 16). Nonetheless, forensic psychiatric patients form a very heterogeneous group regarding psychopathology, criminal history, and risk factors for reoffending (17). This diversity implies that research focusing solely on a single diagnostic group or one medication type is inadequate for drawing definitive conclusions about the significance of treatment persistence for the overall risk of criminal recidivism among forensic psychiatric patients.

### 1.1 Aims of the study

The aim of this study was to investigate post-discharge pharmacological treatment discontinuation in forensic psychiatric patients in Sweden. Specifically, we investigated the prevalence and correlates of discontinuation using nationwide register data.

## 2 Methods

### 2.1 Data

The study cohort included all individuals discharged from forensic psychiatric care between 1 January 2009 and 31 December 2018 who were included in the Swedish National Forensic Psychiatric Register (SNFPR) (*n* = 1,145). SNFRP accounted for 86% of all forensic psychiatric patients in Sweden during this period (18). Data concerning pharmacological treatment were obtained from the Swedish Prescribed Drug Register, which includes all medication dispensations from pharmacies nationwide. Among the included participants, 936 (82%) were male and 209 (18%) were female. Each participant was monitored from the date of discharge from forensic psychiatric services until the end of the follow-up period, which concluded on 31 December 2018. Termination of the follow-up occurred at the date of outcome (medication discontinuation), at the administrative end of follow-up on 31 December 2018, at the administrative end of follow-up 2 years after discharge, date of death, or date of new inpatient episode (psychiatric or somatic) of 30 days or more, whichever transpired first.

### 2.2 Definitions

Medications of the following classes categorized according to the Anatomical Therapeutic Chemical (ATC) classification system were selected: antipsychotics (N05A), antidepressants (N06A), psychostimulants (N06B), drugs used in addictive disorders (N07B), and antiepileptic drugs (N03A). Together, these five categories are referred to as psychotropic medications. As a reference medication, dispensations of any medications used for respiratory organs (R) were used.

Having ongoing pharmacological treatment at the time of discharge was defined as having dispensed medications specified earlier within 90 days of discharge from forensic psychiatric care. A continuous treatment period for each medication was defined based on the assumption that two dispensed prescriptions falling within 90 days of each other belong to the same treatment period. A time frame of 90 days was selected because psychiatric medications are typically not dispensed for more than 90 days at a time in Sweden. This is the so-called “90-day rule”; see, for example, Chang et al. (14). Treatment periods were defined independently of the study follow-up. At the last or single dispensation in a treatment period, treatment end date was estimated by adding 30 days to the date of the latest dispensation. If the estimated end date of the treatment period occurred earlier than the end of follow-up period, the individual was assumed to have discontinued treatment. Data management was executed in SAS, version 9.4, while subsequent analyses were conducted in SPSS, version 26.

#### 2.2.1 Patient variables

Historical, clinical, and situational variables were included in the analyses. Length of stay was presented in years from admission to discharge from forensic psychiatric care. Discharge from Swedish forensic psychiatric care can occur from either inpatient or outpatient care, with discharge from outpatient care being the prevailing norm. However, information on in-/outpatient care before discharge is not included in this dataset, and length of stay refers to the total amount of time in any kind of forensic psychiatric care. Age was computed in whole years during the year of admission and discharge. Pre-index crime substance abuse was defined as having a documented history (does not have to be a diagnosis) of alcohol or other substance abuse as noted in the forensic psychiatric investigation (FPI). Pre-index crime psychiatric care was defined as having engaged with psychiatric in- or outpatient services at least once prior to admission to forensic psychiatric care. Psychiatric diagnoses were categorized based on the initial registration in the SNFPR, based on a chapter in International Statistical Classification of Diseases and Related Health Problems, 10th Revision (ICD-10). The presence of psychosis was defined as having any of the F20 diagnoses as a main or secondary diagnosis. The presence of personality disorder was defined having any of the F60 diagnoses as a main or secondary diagnosis. These broader categories of “psychosis” and “personality disorder” were adopted due to the infrequency of specific diagnoses in this sample, rendering them less practical for analysis. A “limited guardian” (*god man* in Swedish) is someone who receives compensation for overseeing another person’s economic, legal, and personal interests. Having a limited guardian is voluntary. A trustee (*förvaltare* in Swedish) may be appointed against an individual’s wishes, and is only an option when a limited guardian is deemed insufficient (19). Simultaneous appointment of both a trustee and a limited guardian is possible. Supported living accommodation included short-term or permanent accommodation according to the Social Services Act, SoL (20), or the Act Concerning Support and Service for Persons with Certain Functional Impairments, or LSS (21). This variable was recorded at discharge and represented the primary place of living accommodation since previous entry in the SNFPR.

### 2.3 Statistical analyses

Differences between those who did and did not discontinue pharmacological treatment were calculated using a chi-square test for categorical variables and a *t*-test for mean differences for continuous variables. The association between treatment discontinuation and historical, clinical, and situational covariates was estimated with Cox regression analysis separately for each medication type. Bivariate Cox regression analysis was first performed for each patient covariate medication type, after which all covariates with significant associations to discontinuation were entered into a Cox regression model using forward conditional covariate selection. This method involves the stepwise addition of one variable at a time. Variables failing to contribute significantly to the final model are removed, resulting in the best-fitted model as the final model.

## 3 Results

### 3.1 Frequencies of treatment and discontinuation

**Table 1** shows the prevalence of discontinuation for specific medication types, revealing that among the 856 individuals, 488 (57%) discontinued at least one type of psychotropic medication within 2 years post-discharge. The average time to first discontinuation of any treatment with psychotropic medications was 129 days (Md = 76). The most common treatment was treatment with antipsychotics, which 735 (64%) of all discharged individuals had at the time of discharge. Among the individuals undergoing antipsychotic treatment, 45% discontinued this treatment within follow-up; on average 161 days post-discharge (Md =100). Discontinuation rates for other types of medications varied, and 39% discontinued treatment with antidepressants, 62% discontinued treatment with psychostimulants, 74% discontinued treatment with medications used in addictive disorders, and 26% discontinued treatment with antiepileptics. Further specifics are available in **Table 1**.

**Table 1:** The prevalence of pharmacological treatment and treatment discontinuation post-discharge in forensic psychiatric patients with an active dispensation pre-discharge.

Medication at discharge	With pharmacological treatment at discharge	Discontinued medication within 2 years of discharge	Days from discharge to discontinuation for those who discontinued medication within 2 years
	*N* (% of all discharged)	*N* (% of all with treatment)	M (Md)
Any psychotropic drug	856 (75)	488 (57)	129 (76)
Antipsychotics	735 (64)	333 (45)	161 (100)
Antidepressants	361 (31)	141 (39)	159 (90)
Psychostimulants	81 (7)	50 (62)	210 (124)
Drugs used in addictive disorders	93 (8)	69 (74)	113 (68)
Antiepileptics	225 (20)	58 (26)	174 (103)
Drugs used for respiratory organs (control group)	277 (24)	188 (68)	132 (92)

### 3.2 With and without medication

Among individuals discharged from forensic psychiatric care during the period, 75% (856 out of 1,145) were categorized as having ongoing pharmacological treatment at the time of discharge. The individuals with pharmacological treatment were more likely to have received pre-index–crime psychiatric care (*p* = 0.00), to have pre-index–crime substance abuse (*p* = 0.05), to have any form of psychosis (as a primary or secondary diagnosis) (*p* = 0.00), to have a trustee (*p* = 0.00) or a limited guardian (*p* = 0.01) at discharge, and to be living in a supported living accommodation (*p =* 0.00), than those who were not identified as having ongoing pharmacological treatment. Additionally, ongoing treatment was associated with higher age at both admission (*p =* 0.00) and discharge (*p =* 0.00), as well as a longer length of stay (*p =* 0.00). Those without ongoing pharmacological treatment demonstrated a higher likelihood of having a personality disorder (as primary or secondary diagnosis) (*p* = 0.05). See **Appendix 1** for comprehensive details.

### 3.3 Discontinuation and treatment persistence

Among individuals receiving pharmacological treatment, those who discontinued their treatment during the follow-up were less likely to have a trustee (*p* = 0.00), to have a limited guardian (*p* = 0.01), and to be residing in a supported living accommodation (*p* = 0.00) at the time of discharge from forensic psychiatric care, compared to those who continued their pharmacological treatment. Additionally, individuals discontinuing treatment had a shorter length of stay in forensic psychiatric care (*p* = 0.00). See **Table 2** for more details.

**Table 2:** Characteristics in forensic psychiatric patients who did and did not discontinue pharmacological treatment post-discharge.

	Total sample (*n* = 856)	Discontinued treatment (*n* = 488)	Did not discontinue treatment (*n* = 368)	*p* Discontinuation/no discontinuation
	*N* (%)	*N* (%)	*N* (%)	
Women	166 (19)	89 (18)	77 (21)	0.34
Pre-index crime criminal conviction	561 (66)	327 (67)	234 (64)	0.31
Pre-index crime psychiatric care	791 (93)	444 (92)	347 (94)	0.28
Pre-index crime substance abuse	496 (59)	296 (62)	200 (56)	0.10
Index crime violent	735 (86)	422 (87)	313 (85)	0.55
Special court supervision	618 (72)	350 (72)	268 (73)	0.70
Any psychosis	550 (64)	313 (64)	237 (64)	0.94
Any personality disorder	144 (17)	87 (18)	57 (16)	0.41
Psychosis and personality disorder	51 (6)	33 (7)	18 (5)	0.31
Psychosis, no personality disorder	499 (58)	280 (57)	219 (60)	0.58
Personality disorder, no psychosis	93 (11)	54 (11)	39 (11)	0.91
No personality disorder, no psychosis	213 (25)	121 (25)	92 (25)	1.00
Trustee (*förvaltare*) at discharge	171 (20)	64 (13)	107 (29)	0.00
Limited guardian (*god man*) at discharge	162 (19)	77 (16)	85 (23)	0.01
Supported living accommodation at discharge	321 (38)	147 (30)	174 (48)	0.00
	M (Md)	M (Md)	M (Md)	
Age at admission (years)	39.6 (38)	40.1 (39.5)	38.9 (37.5)	0.20
Age at discharge (years)	44.9 (44)	44.6 (44)	45.2 (44)	0.49
Length of stay (years)	5.4 (4.2)	4.6 (3.6)	6.4 (5.0)	0.00

### 3.4 Factors associated with treatment discontinuation

When conducting stepwise forward conditional multivariate Cox regression analyses for individual medication types, Omnibus tests of model coefficients indicated no regression models significantly better than the null model for either psychostimulants (*p* = 0.19) or medications used in addictive disorders (*p* = 0.88). However, having a trustee (HR = 0.35, *p* = 0.03) was associated with a reduced rate of treatment discontinuation for psychostimulants. No single predictor exhibited significant associations with the discontinuation of medications used in addictive disorders.

For the other medication types, the final models (presented in **Table 3**) consistently demonstrated improved explanatory value compared to the null model. Concerning antipsychotics, the presence of both personality disorder and psychosis (compared to psychosis without personality disorder) was associated with an increased rate of treatment discontinuation (HR = 1.52, *p* = 0.04), while having neither a diagnosis of psychosis nor a personality disorder was associated with a decrease in that risk (HR = 0.74, *p* = 0.03). Additionally, factors associated with a lower discontinuation rate was a longer length of stay (HR = 1.00, *p* = 0.00), having a trustee (HR = 0.52, *p* = 0.00), a limited guardian (HR = 0.64, *p* = 0.00), and supported living accommodation (HR = 0.68, *p* = 0.00) at discharge.

**Table 3:** Cox regression analysis of factors associated with pharmacological treatment discontinuation in forensic psychiatric patients in Sweden after discharge from forensic psychiatric care (*n* =856).

	Antipsychotics HR [95% CI]	*p*	Antidepressants HR [95% CI]	*p*	Psychostimulants HR [95% CI]	*p*	Drugs used in addictive disorders HR [95% CI]	*p*	Antiepileptics HR [95% CI]	*p*	Any psychotropic drug HR [95% CI]	*p*	Drugs used for respiratory organs HR [95% CI]	*p*
Being male (female ref.)	0.94 [0.72–1.23]	0.64	0.85 [0.60–1.20]	0.35	1.36 [0.64–2.89]	0.43	1.25 [0.64–2.47]	0.51	1.58 [0.85–2.94]	0.15	0.96 [0.77–1.19]	0.71	0.91 [0.66–1.27]	0.58
Age at discharge	1.01 [1.00–1.01]	0.21	0.99 [0.98–1.00]	0.03	0.97 [0.94–1.01]	0.11	1.01 [0.98–1.03]	0.57	0.99 [0.97–1.01]	0.31	1.00 [0.99–1.01]	0.76	1.00 [0.99–1.01]	0.89
Length of stay (years)	0.95 [0.92–0.98]	0.00	0.91 [0.86–0.96]	0.00	0.98 [0.91–1.04]	0.47	0.97 [0.92–1.03]	0.37	0.88 [0.81–0.95]	0.00	0.95 [0.93–0.97]	0.00	0.97 [0.94-1.00]	0.07
Pre-index crime substance abuse	1.01 [0.82–1.25]	0.90	1.15 [0.83–1.59]	0.39	1.95 [0.92–4.14]	0.08	1.21 [0.52–2.81]	0.65	1.17 [0.73–1.89]	0.51	1.17 [0.98–1.40]	0.08	0.88 [0.65–1.19]	0.39
Pre-index crime psychiatric care	0.74 [0.51–1.07]	0.11	0.48 [0.27–0.86]	0.01	0.24 [0.02–2.36]	0.22	2.02 [0.24–16.95]	0.52	0.95 [0.29–3.15]	0.93	0.84 [0.60–1.16]	0.29	0.33 [0.15–0.69]	0.00
Psychosis/PD		0.01		0.42		0.62		0.62		0.02		0.39		0.04
Psychosis, no PD	Ref.		Ref.		Ref.		Ref.		Ref.		Ref.			
PD, no psychosis	1.10 [0.73–1.64]	0.65	1.32 [0.87–1.99]	0.20	0.60 [0.28–1.29]	0.19	1.29 [0.63–2.64]	0.49	0.95 [0.44–2.08]	0.90	1.19 [0.90–1.58]	0.21	0.93 [0.62–1.38]	0.71
PD and psychosis	1.52 [1.03–2.24]	0.04	1.44 [0.75–2.74]	0.27	1.05 [0.13–8.46]	0.96	1.76 [0.39–8.04]	0.47	2.82 [1.33–5.96]	0.01	1.28 [0.90–1.82]	0.16	2.16 [1.23–3.80]	0.01
Neither psychosis nor PD	0.74 [0.56–0.98]	0.03	0.99 [0.69–1.42]	0.95	0.77 [0.40–1.47]	0.43	0.84 [0.49–1.45]	0.53	1.57 [0.92–2.68]	0.09	1.03 [0.84–1.27]	0.76	1.02 [0.70–1.49]	0.90
Having a trustee	0.52 [0.39–0.70]	0.00	0.65 [0.43–0.98]	0.04	0.35 [0.13–0.91]	0.03	Excl.	Excl0.	Excl.	Excl0.	0.62 [0.49–0.80]	0.00	Excl.	Excl0.
Having a limited guardian	0.64 [0.47–0.87]	0.00	Excl.	Excl0.	Excl.	Excl0.	Excl.	Excl0.	Excl.	Excl0.	0.76 [0.60–0.97]	0.03	Excl.	Excl0.
Supported living accommodation	0.68 [0.54–0.86]	0.00	0.65 [0.43–0.98]	0.00	Excl.	Excl0.	Excl.	Excl0.	0.51 [0.31–0.82]	0.01	0.73 [0.60–0.88]	0.00	0.64 [0.48–0.87]	0.00
−2 Log likelihood	4,415.65	0.00	1,773.18	0.00	396.83	0.19	546.60	0.88	714.94	0.00	6,570.57	0.00	1,961.48	0.00
*N*	710		351		79		89		217		829		268	

Due to the forward conditional selection of included, variables that did not contribute to the final model were excluded.

Regarding antidepressants, higher age at discharge (HR = 0.99, *p =* 0.03), a longer length of stay (HR = 0.99, *p* = 0.00), having a history of pre-index–crime psychiatric care (HR = 0.48, *p* = 0.01), having a trustee (HR = 0.65, *p =* 0.04), and supported living accommodation at discharge (HR = 0.65, *p* = 0.00) were associated with decreased rates of treatment discontinuation.

Concerning antiepileptics, having both a personality disorder and psychosis was associated with an increased rate of discontinuation (HR = 2.82, *p =* 0.01), while longer length of stay (HR = 0.99, *p* = 0.00) and supported living accommodation (HR = 0.51, *p* = 0.01) at discharge were associated with decreased rates of treatment discontinuation.

For any discontinuation of any of psychotropic medication, a longer length of stay (HR = 1.00, *p* = 0.00), having a trustee (HR = 0.62, *p* = 0.00), having a limited guardian (HR = 0.76, *p* = 0.03), and supported living accommodation at discharge (HR = 0.73, *p* = 0.00) were associated with lower rates of treatment discontinuation.

## 4 Discussion

The results showed varying discontinuation rates of pharmacological treatment with psychotropic medications, ranging from 26% (antiepileptic drugs) to 74% (drugs used in addictive disorders) within the 2-year post-discharge period. The most prevalent type of medication at discharge was antipsychotics, with 45% of individuals on antipsychotic treatment discontinuing it during follow-up—a rate comparatively lower than previously observed regarding the treatment persistence of antipsychotic medications among non-forensic patients (6). The group of individuals without pharmacological treatment at discharge differed from the group with pharmacological treatment regarding several background factors, clinical factors, and historical factors. Moreover, within the cohort of individuals with pharmacological treatment, those discontinuing any treatment were less likely to have supportive measures such as a trustee, a limited guardian, or a supported living accommodation in place at discharge.

When investigating factors associated with discontinuation across distinct psychotropic medications, a longer length of stay, having a trustee, having a limited guardian, and living in a supported living accommodation were associated with treatment persistence across multiple medication types. These differences persisted even after adjusting for age, sex, diagnostic differences, and history of substance abuse. Consistent with prior conclusions (4, 22), numerous factors likely contribute to medication discontinuation. Notably, within this cohort, factors of importance for treatment persistence appear related to higher levels of individual support, aligning with previous research emphasizing the significance of social and environmental risk factors—such as the individual’s support structure—for medication adherence (9). Factors related to different types of support structures have also demonstrated an association with a reduced likelihood of criminal reconviction (23). While this study does not address the correlation between treatment discontinuation and criminal recidivism, it underscores the role of higher levels of individual support post-discharge for this group. The inquiry into the potential association between treatment discontinuation and criminal recidivism is, however, highly relevant for clinicians in and out of forensic psychiatry and necessitates future investigation.

### 4.1 Strengths and limitations

This study has limitations concerning categorization, for example, concerning the categorization of diagnoses. It is possible that the use of more specific diagnostic categories (for example, bipolar disorder) would have yielded more precise outcomes. However, the adoption of broader diagnostic categories in this study aimed to serve as indicators of symptoms rather than of specific diagnoses. Employing more specified categories could also have shown more specific results regarding types of medications. For instance, categorizing “Antipsychotics” into long-acting injectable antipsychotics versus oral forms might have shown differences in treatment persistence not accounted for in this study. Future research could address these aspects more in depth, providing a more nuanced exploration.

The Prescribed Drug Register does not include medications administered within hospital settings. This absence is likely to affect the estimated prevalence of medication use at discharge in this study. It remains uncertain whether individuals not identified as having an active pharmacological treatment are truly not prescribed any medications or receive them through hospital administration. During forensic psychiatric care, 94% of patients are undergoing pharmacological treatment with a psychotropic medication (24), offering an approximate gauge of the underestimation in this study’s findings, even if the exact proportion is likely to be slightly different at the time of discharge. The omitted subgroup likely differs systematically from those who are included in the Prescribed Drug Register, which means that the results have to be interpreted cautiously. Consequently, these findings might not be generalizable to all patients discharged from forensic psychiatric care but rather to those acquiring medications via personal dispensation rather than through hospital channels. However, the results do provide novel insights about the former forensic psychiatric patients obtaining medications outside hospital settings post-discharge, a group that consists of the majority of discharged individuals. The utilization of registry data spanning a decade ensures robust data quality.

Given the limited existing research on treatment persistence and discontinuation among former forensic psychiatric patients, this knowledge is especially important, as forensic psychiatric patients are a distinct cohort, making it unwise to extrapolate findings from other groups to this specific population. Moreover, the sample size in this study is a notable strength. Research involving forensic psychiatric patients often suffers from small sample sizes, a concern that has been highlighted by several researchers (25–27).

## 5 Conclusion

Several situational factors are linked to pharmacological treatment persistence among former forensic psychiatric patients. These factors include having a trustee, having a limited guardian, and residing in a supported living accommodation, indicating high levels of individual support given to these individuals. This insight holds significance for professionals who are involved in pre-discharge planning within forensic psychiatric care and those who interact with this cohort of former patients post-discharge.

## Data availability statement

Data are available from the registers that are included in the study. Since the registers are not publicly available, restrictions regarding the availability of the data apply. However, upon reasonable request and with permission of the included registers data are available from the authors. Requests to access these datasets should be directed to Swedish National Forensic Psychiatric Register, helena.i.andreasson@skane.se. National Prescribed Drug Register, E-post: inrapportering@socialstyrelsen.se.

## Author contributions

EN: Conceptualization, Data curation, Formal Analysis, Methodology, Writing – original draft. SV: Conceptualization, Formal Analysis, Methodology, Writing – review & editing. FK: Conceptualization, Writing – review & editing. ZC: Supervision, Writing – review & editing. MS: Supervision, Writing – review & editing.

## Appendix I Characteristics in forensic psychiatric patients with and without an active medication* dispensation pre-discharge.

**Table:**

	With medication, (*n*=856)	Without medication (*n*=289)	Total sample (*n*=1145)	*P* With and without medication
	*N (%)*	*N (%)*	*N (%)*	
Women	166 (19)	43 (15)	209 (18)	.09
Pre-index crime criminal conviction	561 (66)	175 (61)	736 (64)	.14
Pre-index crime psychiatric care	791 (93)	237 (84)	1028 (91)	.00
Pre-index crime substance abuse	496 (59)	148 (53)	644 (58)	.05
Index crime violent	735 (86)	247 (86)	982 (86)	.85
Special court supervision	618 (72)	221 (77)	839 (73)	.17
Any psychosis	550 (64)	132 (46)	682 (60)	.00
Any personality disorder	144 (17)	64 (22)	208 (18)	.05
Psychosis and PD	51 (6)	9 (3)	60 (5)	.07
Psychosis, no PD	499 (58)	123 (43)	622 (54)	.00
PD, no psychosis	93 (11)	55 (19)	148 (13)	.00
No PD, no psychosis	213 (25)	102 (35)	315 (28)	.00
Trustee (förvaltare) at discharge	171 (20)	27 (9)	198 (17)	.00
Limited guardian (god man) at discharge	162 (19)	35 (12)	197 (17)	.01
Supported living accommodation at discharge	321 (38)	43 (15)	364 (32)	.00
	***M (Md)***	***M (Md)***	***M (Md)***	
Age at admission (years)	39.6 (38)	36.3 (33)	38.8 (37)	.00
Age at discharge (years)	44.9 (44)	40 (37)	43.6 (42)	.00
Length of stay (years)	5.3 (4.2)	3.7 (2.8)	4.9 (3.7)	.00

*According to this study’s method for selection.
